# An Evolutionarily Conserved Structural Platform for PRC2 Inhibition by a Class of Ezh2 Inhibitors

**DOI:** 10.1038/s41598-018-27175-w

**Published:** 2018-06-14

**Authors:** Matthew Bratkowski, Xin Yang, Xin Liu

**Affiliations:** Cecil H. and Ida Green Center for Reproductive Biology Sciences and Division of Basic Research, Department of Obstetrics and Gynecology, Department of Biophysics, UT Southwestern Medical Center, Dallas, TX 75390 USA

## Abstract

Polycomb repressive complex 2 (PRC2) mediates trimethylation of histone H3K27 (H3K27me3), an epigenetic hallmark for repressed chromatin. Overactive mutants of the histone lysine methyltransferase subunit of PRC2, Ezh2, are found in various types of cancers. Pyridone-containing inhibitors such as GSK126 compete with S-adenosylmethionine (SAM) for Ezh2 binding and effectively inhibit PRC2 activity. PRC2 from the thermophilic fungus *Chaetomium thermophilum* (*ct*) is functionally similar to the human version in several regards and has the added advantage of producing high-resolution crystal structures, although inhibitor-bound structures of human or human/chameleon PRC2 are also available at up to 2.6 Å resolution. We solved crystal structures of both human and *ct*PRC2 bound to GSK126 and the structurally similar inhibitor GSK343. While the two organisms feature a disparate degree of inhibitor potency, surprisingly, GSK126 binds in a similar manner in both structures. Structure-guided protein engineering of the drug binding pocket allowed us to introduce humanizing mutations into *ct*Ezh2 to produce a *ct*PRC2 variant that is more susceptible to GSK126 inhibition. Additional analysis indicated that an evolutionarily conserved structural platform dictates a unique mode of GSK126 binding, suggesting a mechanism of drug selectivity. The existing drug scaffold may thus be used to probe the function and cellular regulation of PRC2 in a wide spectrum of organisms, ranging from fungi to humans.

## Introduction

Polycomb Repressive Complex 2 (PRC2) is a histone methyltransferase complex composed of core subunits Ezh2 (or Ezh1), Eed, Suz12 and Rbbp4. PRC2 trimethylates histone H3K27 (H3K27me3) to mark developmentally repressed chromatin. Either Ezh1 or Ezh2 can serve as the methyltransferase for PRC2, but only Ezh2 is associated with actively proliferating cells^1^. The PRC2 core complex is also regulated by a number of accessory subunits during distinct stages of development^2,3^.

Ezh2 contains a split catalytic region composed of the SET domain and the SET Activation Loop (SAL) but is inactive without minimally associating with Eed and the VEFS domain of Suz12 [Suz12(VEFS)]^4–8^. PRC2 uses S-adenosylmethionine (SAM) to methylate unmethylated (me0) and monomethylated (me1) H3K27 but displays little activity on dimethylated H3K27 (me2)^9^. The H3K27me3 end product binds to Eed to cause a conformational change of the Stimulation Response Motif (SRM) of Ezh2, which results in stimulation of the enzymatic activity of PRC2 that may promote formation of large repressive H3K27me3 chromatin domains^4,10,11^.

PRC2 has several connections to cancer. Ezh2 is overexpressed in breast, prostate, and liver cancer among others^12^. Point mutations in Ezh2 also alter the substrate preference and product specificity of PRC2 in Non-Hodgkin lymphomas (NHLs). Mutations of the SET domain residue Y641 result in increased activity of PRC2 on H3K27me2 substrates and greatly reduced activity on H3K27me0 substrates^13–15^. In contrast, the A677G mutation results in an almost equal preference of PRC2 for all the methylation states^9^. Furthermore, the A687V mutation exhibits decreased activity on H3K27me0 and dramatically increased activity on H3K27me1 substrates^16^. Remarkably, the histone H3 mutant H3K27M is found in pediatric glioblastoma to bind tightly to Ezh2, and prevents establishment of a normal H3K27me3 program in cells^17,18^.

**Figure 1:**
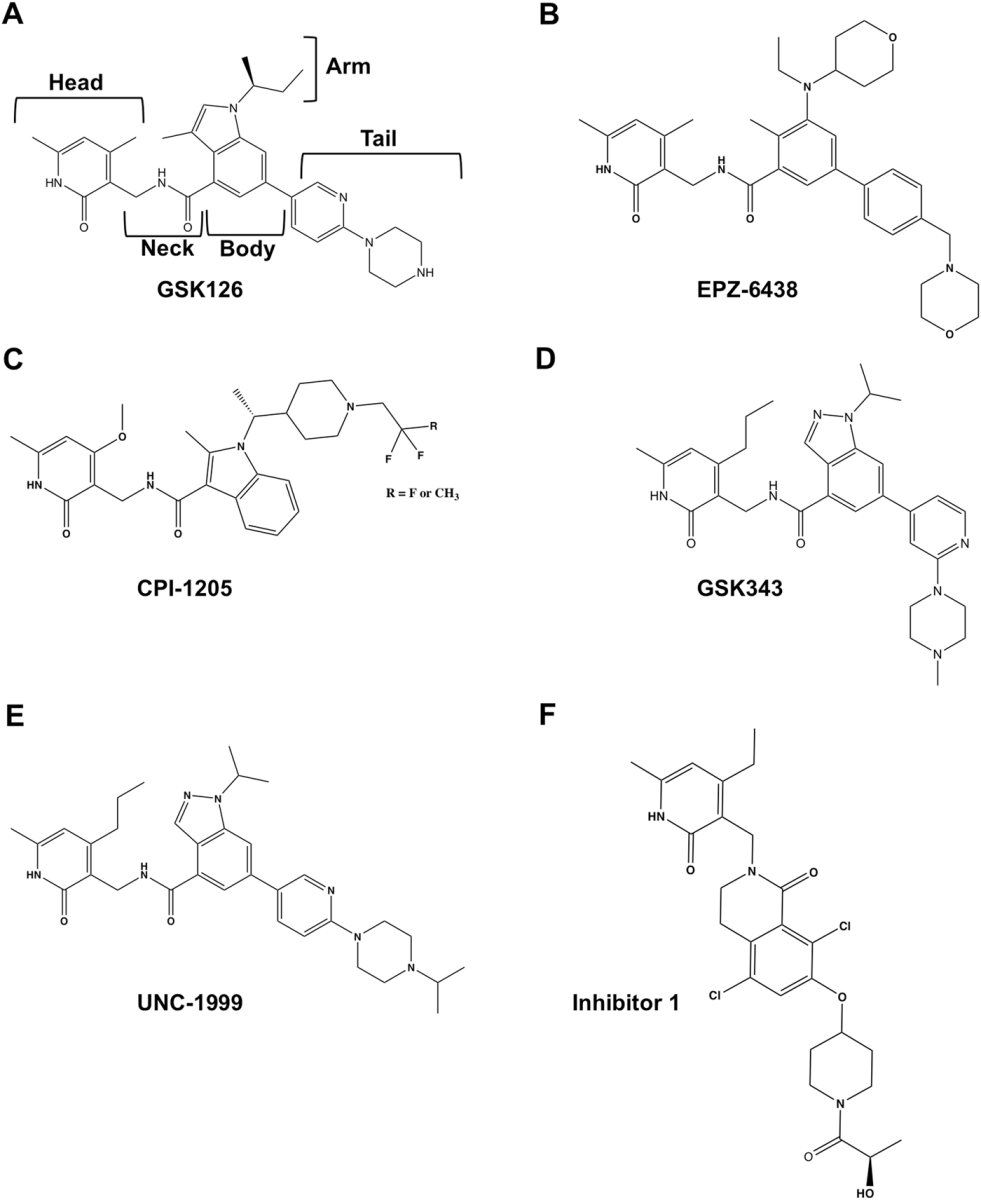
Structures of pyridone inhibitors. General regions of the drug are labeled for GSK126. This figure was prepared using ChemDraw software (Perkin Elmer).

PRC2 has become a popular target for cancer therapeutics. Most PRC2 inhibitors feature a pyridone moiety as the main pharmacophore that is a SAM competitive inhibitor (Fig. 1). They feature a fairly similar pyridone “head” region, an amide linker “neck” region, a variable aromatic “body” region (indole, indazole, or another hydrophobic group), and variable “arm” and “tail” structures attached to the top and bottom of the body, respectively (Fig. 1, *labeled for GSK126*). Pyridone inhibitors include GSK126 (Fig. 1A), EPZ-6438 (Tazemetostat) (Fig. 1B), and CPI-1205 (Fig. 1C) that are effective in suppressing tumor growth and are in clinical trials as cancer therapeutics^19–22^. Pre-clinical pyridone compounds including GSK343 (Fig. 1D), UNC-1999 (Fig. 1E), and the lactam analogue “inhibitor 1” (Fig. 1F) also inhibit PRC2^23–25^. Pyridone inhibitors display varying degrees of selectivity for PRC2 containing Ezh2 over Ezh1 (96% identical to the SET domain of Ezh2) with multiple functional groups impacting selectivity^26^.

**Figure 2:**
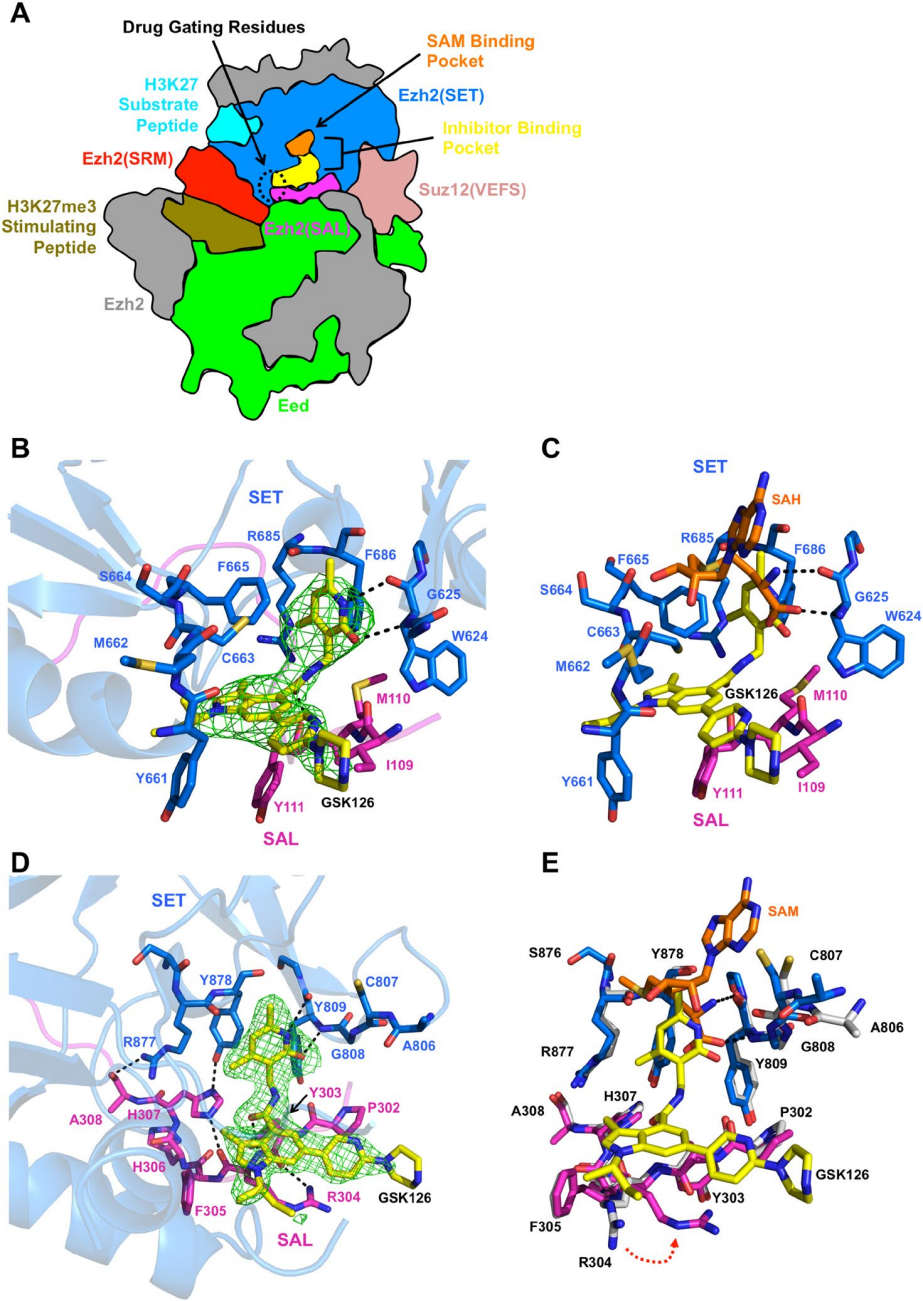
Structures of *ct*PRC2 bound to pyridone inhibitors. (**A**) Schematic of PRC2 with important regions color-coded. This illustration is based on surface representations of the human complex in the stimulated state (PDB 5hyn) and the *h/Ac* PRC2 bound to inhibitor 1 structure (PDB 5ij7). (**B**) Structure of human PRC2 in complex with GSK126 (*yellow sticks*). The SET and SAL domains are colored in blue and magenta sticks, respectively. Relative orientations of the drug-binding pocket (*top*, *right and bottom*, *left*) are labeled. Fo − Fc difference electron density is shown as green mesh and is contoured at 2.5σ. Black, dashed lines indicate hydrogen bonds. This figure, and all other protein structure figures in the manuscript, was drawn with PyMOL software (The PyMOL Molecular Graphics System, Version 1.8 Schrödinger, LLC). (**C**) Alignment of the GSK126-bound human PRC2 structure with the human PRC2/SAH/K27M/K27me3 structure (PDB 5hyn) indicates that the pyridone region of GSK126 partially overlaps with SAH/SAM binding. (**D**) Structure of *ct*PRC2 bound to GSK126. The drug-binding pocket formed by the SET (*blue*) and SAL (*magenta*) domains of Ezh2 is shown in sticks, with residues labeled. GSK126 (*yellow sticks*) is shown inside of Fo − Fc difference electron density (*green mesh*) contoured at 3σ. Hydrogen bonds are depicted as black, dashed lines. (**E**) GSK126 partially overlaps with the SAM binding site. Alignment of GSK126 bound *ct*PRC2 (with SET and SAL domains color-code as above) with the stimulated state *ct*PRC2 structure (*gray sticks*, PDB 5kkl). SAM and GSK126 are in orange and yellow sticks, respectively. Residue R304 undergoes a rotameric conformational change from the SAM-bound to GSK126 bound structures (*red*, *dotted arrow*).

Pyridone inhibitors bind to Ezh2 in an extended pocket that overlaps with the SAM binding site (Fig. 2A). The pocket is enclosed by a “gating region” formed by a unique interface of the SET and SAL that borders Eed (Fig. 2A). Although promising as therapeutics, pyridone inhibitors are ineffective for acquired Ezh2 mutations at drug gating residues Y661, Y111, and I109^27,28^. Crystal structures of a human/American chameleon (*h/Ac*) PRC2 bound to inhibitor 1 (at 2.62 Å resolution) and a human PRC2 bound to a CPI-1205 derivative (CPI-1205d) (at 3.47 Å resolution) show that the body and variable elongated arm regions of the drugs are caged by Ezh2 residues Y661 and Y111, which helps to explain why mutation at these sites results in loss of drug potency^21,27–29^.

PRC2 components are present in many eukaryotes yet are absent in yeasts *S*. *cerevisiae* and *S*. *pombe*^2^. However, PRC2 is required for H3K27 methylation in the bread mold *Neurospora crassa*, the wheat and barley pathogen *Fusarium graminearum*, and the human pathogen *Cryptococcus neoformans*^30–32^. Recent crystal structures of PRC2 from the thermophilic yeast *Chaetomium thermophilum* (herein referred to as “*ct*”) provided a useful model of the prototypical fungal PRC2^4,33^. The SAM binding pocket of *ct*Ezh2 is structurally similar to the human version, but the drug gating residues in the SAL and SET of human Ezh2 that are necessary for pyridone inhibitor potency^27,28^ do not appear to have homologues in fungal Ezh2 based on sequence analysis (Fig. S1). Thus, it is unclear whether pyridone inhibitors can interact with fungal Ezh2 in a manner similar to the human version.

In order to gain a more detailed understanding of the interaction of PRC2 with pyridone inhibitors and extend the existing drug scaffold to the study of fungal PRC2, we solved the crystal structure of human and *ct*PRC2 bound to GSK126. Although GSK126 binds to *ct*PRC2 in a similar manner to human PRC2 despite limited sequence conservation in the drug-binding site (Fig. S1 and Fig. 2A), it is a poor inhibitor of *ct*PRC2. However, humanizing mutations increased GSK126 inhibition and allowed us to solve additional structures of humanized *ct*PRC2 bound to GSK126 and GSK343 that revealed drug interactions for a model more similar to human PRC2 at high resolution. Overall, these studies demonstrated increased diversity among the binding modes of pyridone inhibitors to PRC2 and provided feasibility for the study of PRC2 function in multiple organisms by chemical genetics.

## Results

### Structures of human and ctPRC2 bound to GSK126

To obtain a more holistic understanding of PRC2 inhibition by pyridone inhibitors, we attempted to solve crystal structures of human PRC2 in complex with inhibitors that had not been previously characterized. We established a system to co-express a fusion of full length Ezh2 and [Suz12(VEFS)] with full length Eed in *S*. *cerevisiae*, and further engineered Ezh2 by deleting amino acids 183–195 and 341–427 to produce diffracting crystals (protein purity in Fig. S2A). While several pyridone inhibitors were attempted for structural analysis, we were only able to obtain a co-crystal structure of human PRC2 bound to GSK126 (statistics in Table S1).

Although the resolution of the crystal structure is limited to 3.9 Å, F_o_ − F_c_ difference electron density for GSK126 was clear enough to build the drug into the structure (Fig. 2B). Continuous electron density is observed for the body of the drug, while the tail, arm, and head regions are partially resolved to varying degrees (Fig. 2B and Fig. S3). We note that there is some ambiguity regarding the absolute conformations of terminal regions of the drug in this structure as well as the other structures discussed in this report (see below). Electron density for residues around the drug-binding site was of sufficient quality to assign some side chains as well (Fig. S3). The top, right-hand side of the drug-binding pocket (Fig. 2B, *relative to the pyridone moiety at top*) is formed by residues W624, R685, and F686. In a manner similar to other pyridone inhibitors^21,29^, the amide nitrogen and carbonyl oxygen of the pyridone head of GSK126 mediate distorted hydrogen bonds with the main chain carbonyl oxygen and amide nitrogen, respectively, of residue W624 of Ezh2 to compete with SAM binding to the same residue (Fig. 2B,C). Residues I109, M110 and Y111 of the SAL of Ezh2 form the bottom of the binding pocket (Fig. 2B). The backbone carbonyl oxygen on the neck region between the pyridone and the indole moieties of the drug binds to the backbone amide nitrogen of residue Y111, and the side chain of Y111 interacts with the indole body moiety (Fig. 2B). The left-hand side of the pocket is formed by residues 661–665 of the SET domain (Fig. 2B), and this region constricts access to the pocket (Fig. S4A). Residues Y661 and Y111 form a gating region in a manner similar to other PRC2 structures (Fig. 2B). One feature that appears unique to GSK126 is that the linked pyridine and piperazine rings of its tail region jut out above the gate toward the solvent (Fig. 2B and Fig. S4A).

While the human GSK126-bound PRC2 structure revealed interesting new aspects of drug binding, the low resolution of the structure limited more in-depth analysis. Although structures of human or human/chameleon PRC2 bound to certain inhibitors have been reported^21,29^, efforts to improve the structure resolution of PRC2 bound to other inhibitors such as GSK126, however, were unsuccessfully. Previous work from our lab produced high-resolution crystal structures of *ct*PRC2^4,33^, indicating that it can serve as a useful model for determining fine molecular details of the complex. We thus sought to use *ct*PRC2 in structural studies with GSK126 to determine whether recognition was conserved between human and fungal species, and if so, to obtain a higher resolution model of PRC2 bound to GSK126.

We solved the co-crystal structure of *ct*PRC2 bound to GSK126 to 2.5 Å resolution (crystallization statistics in Table S1 and protein purity in Fig. S2A). Overall, the complex assumes an autoinhibited conformation in a manner similar to the apo complex^33^. The hydrophobic drug-binding pocket of Ezh2 is formed by residues P302, R304, F305 and H307 in the SAL domain, and Y809 and Y878 in the SET domain (Fig. 2D, *magenta and blue sticks*, *respectively*). Clear electron density was present for GSK126 in all regions except the *sec*-butyl “arm” group and the terminal piperazine ring of the “tail” (Fig. 2D and Fig. S5A), even though the drug molecule binds in a site that is less enclosed by the *ct*Ezh2 residues compared to the human structure (Fig. 2B,D and Fig. S4B). The carbonyl and amine groups of the pyridone moiety of GSK126 mimic the carboxylate and amine of SAM to hydrogen bond to the backbone of Y809, directly competing with SAM binding in a manner similar to the human version (Fig. 2C,E).

In the SAL domain, the imidazole nitrogen of residue H307 hydrogen bonds to the carbonyl on the linker between the pyridone and the indole moieties of the drug, and residue H307 itself is stabilized by hydrogen binding to the carbonyl oxygen of residue R304 (Fig. 2D). The backbone amine of residue R304 also hydrogen bonds to the linker carboxyl oxygen in the “neck” region of the drug, whereas the side-chain of residue R304 undergoes a conformational change compared to the SAM-bound structure (Fig. 2E), and supports both the indole region of the body and the pyridine region of the tail of the drug via hydrophobic interactions. The side chain of residue P302 also provides additional support for the latter. The piperazine region points outward toward the solvent, similar as to the GSK126-bound human PRC2 structure (Fig. 2B). Thus, our structure indicates that pyridone inhibitors can also bind *ct*PRC2, despite both sequence and structural differences in the drug binding pocket between human and *ct*PRC2.

### Comparison of the inhibition of human and *ct*PRC2 by GSK126

**Figure 3:**
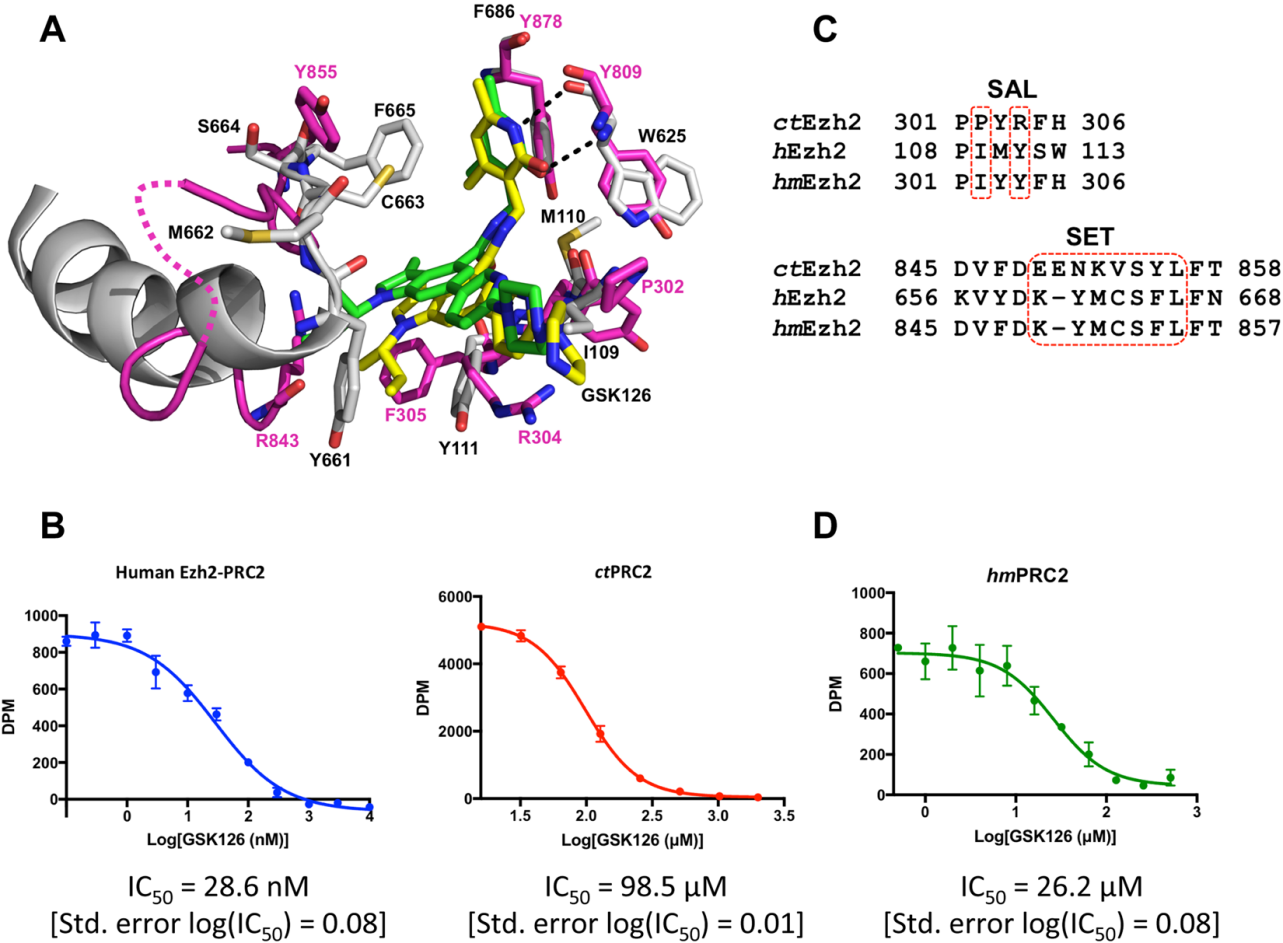
Comparison of *ct*PRC2 and human PRC2-GSK126 bound structures. (**A**) Alignment of Ezh2 from *ct*PRC2 (*magenta*) and human (*gray*) PRC2-GSK126 bound structures. The GSK126 molecule from the *ct*PRC2 structure is in yellow sticks, and that from the human PRC2 structure is in green sticks. A disordered region of *ct*Ezh2 is represented by a dotted line. (**B**) Dose-response fitting curves for human PRC2 *(left*) and *ct*PRC2 (*right*) under a titration of GSK126. IC_50_ values are indicated below the fitting curves (note that they were measured under different SAM concentrations). “DPM” indicates disintegrations per minute read-out from scintillation counting. Error bars represent standard deviation of two replicates. Error in log(IC_50_) represents the standard error. (**C**) Location of humanizing (*hm*) mutations in the SAL (*top*) and SET (*bottom*) domains of *ct*Ezh2 that composed *hm*Ezh2. Wild-type human (*h*) and *ct*Ezh2 and shown for comparison. Note that the numbering scheme of the SET domain accounts for the fact that *hm*PRC2 is shortened by one residue compared to *ct*PRC2. (**D**) Dose-response fitting curve for *hm*PRC2 under a titration of GSK126, with the IC_50_ value listed below the curve. Error bars represent the standard deviation of two replicates. Error in log(IC_50_) value represents the standard error.

Structural alignment indicates that GSK126 binds to a similar composite surface formed by the SAL and SET domains of Ezh2 in both human and *ct*PRC2 (Fig. 3A), despite the notable sequence divergence in these two domains (Fig. S1). Functional roles of residues in *ct*PRC2 for GSK126 binding are not easily identified based on the primary sequence alignment. For instance, *ct*Ezh2 residue R304 instead of residue F305 mediates a similar drug interaction as residue Y111 in human Ezh2 (Fig. 3A), despite the fact that the latter two align better on a primary sequence level (Fig. S1A). Additionally, *ct*Ezh2 residue P302 stabilizes the tail region of the drug in a manner similar to residue I109 in human Ezh2 (Fig. 3A). Thus, the drug-gating residues in the SAL domain have a similar function in *ct*Ezh2 despite limited sequence conservation to human Ezh2. Notably, the conformations of the bound drug molecules in human and *ct*PRC2 also well resemble each other (Fig. 3A).

While GSK126 is a potent inhibitor of human PRC2, it is unclear whether the drug inhibits fungal PRC2 complexes in a similar fashion, and we tested this possibility. We first established that a minimal human PRC2 [Ezh2-Eed-Suz12(VEFS)] similar in composition to the crystallized *ct*PRC2 was inhibited by GSK126 with an IC_50_ value of about 30 nM (Fig. 3B, and protein purity in Fig. S2A). This value is higher than the reported IC_50_ value of 12 nM for the five-subunit (Ezh2, Suz12, Eed, Rbbp4, Aebp2) complex^19,34^, and we note that the difference could be caused by the lack of additional subunits. We then tested the inhibition of *ct*PRC2 by GSK126. In contrast to human PRC2, GSK126 was a very poor inhibitor of *ct*PRC2 with an IC_50_ value of about 100 μM (Fig. 3B, and protein purity in Fig. S2A).

Likely, poor structural conservation in certain regions of the SET domain contributes to the disparate drug properties of GSK126 for human and *ct*PRC2. While residues 656–661 fold into a tight helix in human Ezh2 and residue Y661 cages the drug in the active site, the aligned region in *ct*Ezh2 (residues 845–851) contains a partially disordered flexible loop (Fig. 3A, *dotted line*), that is located away from the drug. This causes *ct*Ezh2 to contain a relatively open drug-binding pocket compared to the constricted pocket found in human Ezh2 (Fig. S4B compared to Fig. S4A).

Based on our structural analysis, we introduced several “humanizing” mutations (*hm*) into the SET and SAL domains of *ct*Ezh2 (summarized in Fig. 3C). Specifically, *hm*Ezh2 contains a SAL domain with P302I and R304Y mutations to mimic the human counterpart (Fig. 3C, *top alignment*). Another edited region was the loop composed of residues 849–855. Notably, this region is poorly conserved with the corresponding aligned region of human Ezh2 that directly mediates drug binding and contains one of the gating residues, Y661 (Fig. 3C, *bottom alignment*). This region was mutated to resemble residues of the human Ezh2, under the assumption that this may result in a tighter drug-binding pocket. In enzyme kinetic analysis, *hm*PRC2 had overall comparable Km and kcat for the histone peptide substrate as wild-type complex (Fig. S6). In IC_50_ analysis with purified protein complex and GSK126, *hm*PRC2 had an IC_50_ value that was about four-fold lower than wild-type *ct*PRC2, (Fig. 3D, compare to Fig. 3B; protein purity in Fig. S2A). While this value is still much higher than that of human PRC2, it indicates that humanizing mutations improve drug potency, probably due to increased drug binding affinity.

### Structures of Humanized *ct*PRC2 bound to GSK126 and GSK343

**Figure 4:**
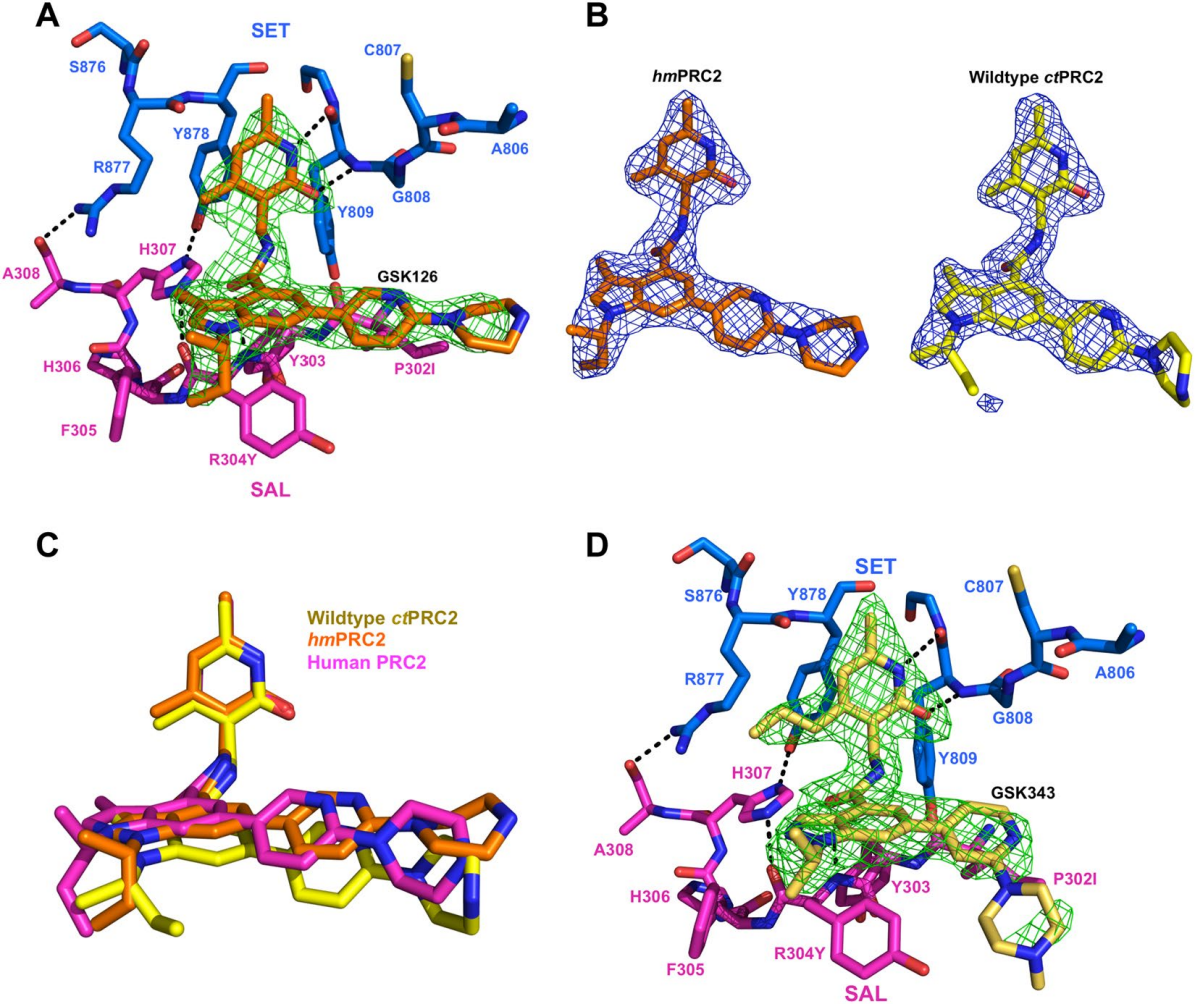
Structure of humanized *hm*PRC2 bound to pyridone inhibitors. (**A**) Structure of *hm*PRC2 bound to GSK126. SET and SAL domains are in blue and magenta sticks, respectively. GSK126 is in orange sticks. Green mesh represents Fo − Fc difference electron density contoured at 3σ. (**B**) GSK126 molecule from *hm*PRC2 bound structure (*left*, *orange sticks*) and wild-type *ct*PRC2 bound structure (*right*, *yellow sticks*). Molecules are enveloped by 2Fo − Fc electron density contoured at 1σ (*blue mesh*). (**C**) Structural alignment of GSK126-bound PRC2 structures. GSK126 molecules are shown as sticks and are color-coded based on the corresponding structure. (**D**) Structure of *hm*PRC2 bound to GSK343. Domain color-coding follows the scheme in Fig. 4A. GSK343 (*tan sticks*) is enclosed by Fo − Fc difference electron density contoured at 3σ (*green mesh*).

We next sought to solve the crystal structure of *hm*PRC2 bound to GSK126 to determine whether the humanizing mutations actually resulted in a tighter drug-binding pocket on the molecular level. The co-crystal structure of GSK126-bound *hm*PRC2 was solved at 2.65 Å resolution (Fig. 4A and statistics in Table S1). Despite a lower overall resolution compared to the wiltype structure, the *hm*PRC2 structure exhibits an improved electron density map for GSK126 that enclosed the entirety of the drug compared to the wild-type complex (Fig. 4B, *compare hmPRC2 on left to ctPRC2 on right*, and Fig. S5B). The body and tail of the inhibitor in *hm*PRC2 are shifted to a location that is akin to the human PRC2-GSK126 structure (Fig. 4C). The P302I and R304Y mutations more closely approach the drug and apparently provide improved stability for the pyridine-piperazine tail of GSK126 (Fig. 4A, and detailed in Fig. S5B). Thus, humanizing mutations in the SAL domain likely contribute to the enhanced inhibition of the enzyme.

*hm*PRC2 contained replacements of residues 849–855 of the SET domain with human residues in attempts to impose an improvement of drug binding (Fig. 3C). However, electron density is largely missing for this region in the *hm*PRC2 structure, implying that it did not fold in the same manner as the *bona fide* human version, at least in the current format (Fig. S5A, *compared to* Fig. S5B). Mutations instead caused *hm*PRC2 to form a more open drug-binding pocket than *ct*PRC2 (Fig. S4C, compared to Fig. S4B). However, it should be noted that additional electron density was apparent for the *sec*-butyl arm of GSK126 in the *hm*PRC2 structure that was missing in the *ct*PRC2 version (Fig. 4B and Fig. S5B).

We were also able to determine the structure of *hm*PRC2 in complex with GSK343 and an H3K27me3 stimulating peptide at 2.3 Å resolution. Overall, GSK343 is recognized in a similar manner as GSK126 (Fig. 4D). Additional electron density is present for the longer propyl group, but there is a lack of density for the piperazine region of the tail, possibly because it bends in a different direction compared to GSK126 (Fig. 4D and Fig. S5C). Despite the structure being at a higher resolution than the wild-type *ct*PRC2-GSK126 or *hm*PRC2-GSK126 complexes, the humanized loop region of the SET domain is still largely disordered (Fig. S5C, *compared to S5A*). Notably, GSK343 is also a poor inhibitor of *ct*PRC2 but produced an IC_50_ value at least six-fold lower for *hm*PRC2 compared to wild type *ct*PRC2 in a manner similar to that observed for GSK126 **(**Fig. S7**)**. Interestingly, while the H3K27me3 peptide clearly binds to the aromatic cage on Eed, the SRM domain of Ezh2 remains disordered (Fig. S8).

### Comparison of the structures of human PRC2 bound to GSK126 and other pyridone inhibitors

**Figure 5:**
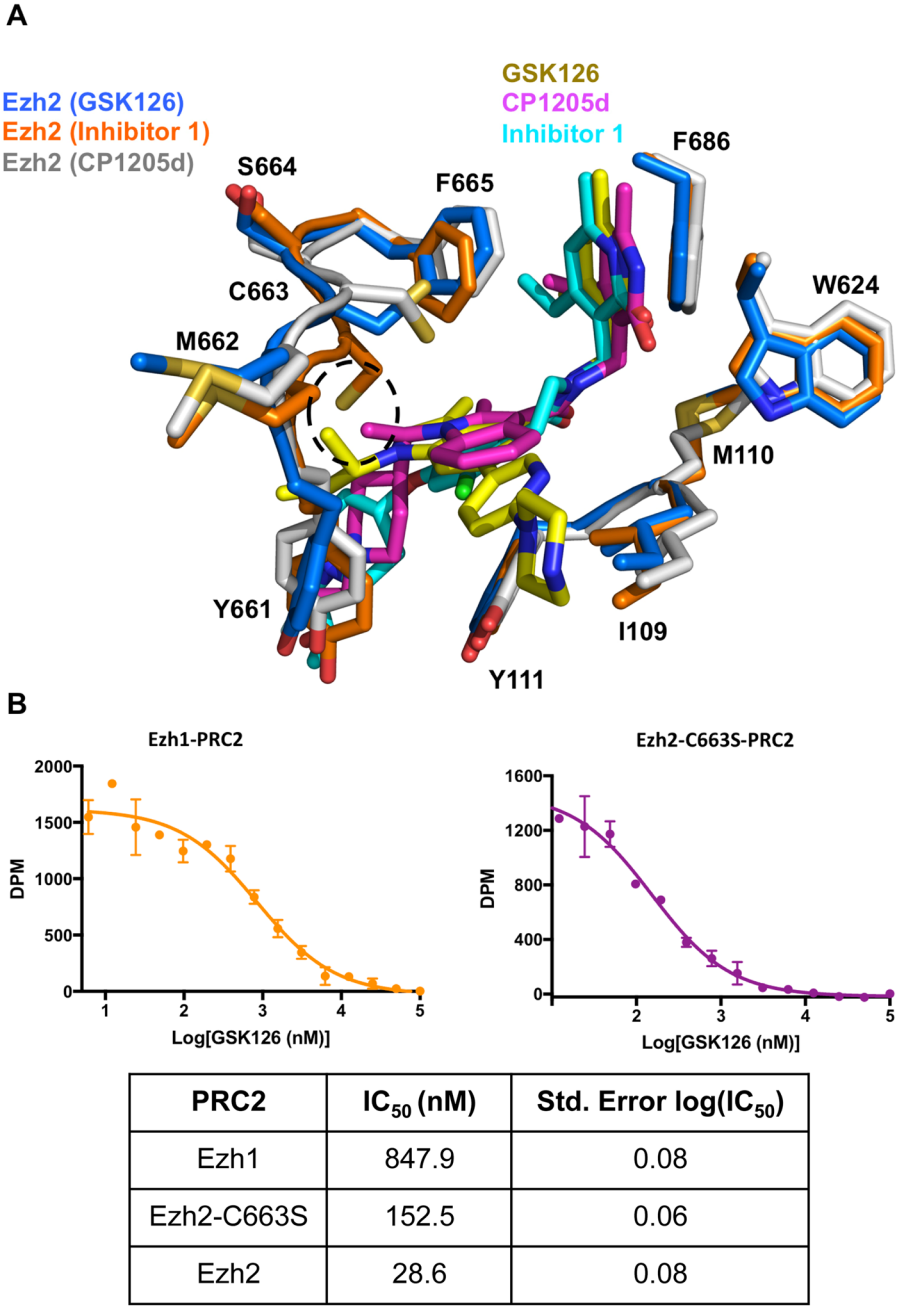
Comparison of human GSK126-bound PRC2 with other inhibitor bound structures. (**A**) Structural alignment of human GSK126-bound Ezh2 (*blue sticks*) with the CPI-1205d bound structure (PDB 5ls6, *gray sticks*) and the inhibitor 1 bound structure (PDB 5ij7, *orange sticks*). Inhibitors are shown as sticks and are color-coded. (**B**) Dose-response fitting curves of Ezh1-PRC2 (*left*, *orange*) and Ezh2-C663S-PRC2 (*right*, *purple*) under a titration of GSK126. IC_50_ values are in the table below the curves, and the value of Ezh2 (from Fig. 3B) is included for direct comparison. Error bars represent the standard deviation of two replicates. Error in log(IC_50_) value represents the standard error.

The structures of *hm*PRC2-GSK126 and *hm*PRC2-GSK343 with an improved resolution revealed an overall similar drug conformation as our human structure. We next sought to compare the structure of GSK126-bound human PRC2 with previous structures of human or human/chameleon PRC2 bound to other pyridone inhibitors. While the SAM-competing pyridone region of GSK126 interacts with Ezh2 in the same manner as other inhibitors, the placement of other functional groups of GSK126 within the drug-binding pocket differs (Fig. 5A). GSK126 features an extended pyridine-piperazine tail moiety, in contrast to CPI-1205d and inhibitor 1 that lack tail moieties (Fig. 1A, *compare with* Fig. 1C,F). In the crystal structure, the linked pyridine and piperazine rings of GSK126 point outward toward the solvent in a distinct manner (Figs 5A and 2D). The pyridine ring is supported by a hydrophobic interaction with I109 (Fig. 5A), and an I109K mutation results in loss of drug potency^27,28^.

GSK343, UNC1999, and EPZ-6438 all feature long tail moieties (Fig. 1D,E and B, *respectively*), which are potentially solvent exposed when bound to PRC2 similar to GSK126. Comparison with the GSK343-bound *hm*PRC2 structure suggests that the tail of GSK343 is likely pointed outward when bound to human PRC2 (Fig. 4D compare to Fig. 2B). Notably, a recent docking study of UNC1999 postulated that its tail would be positioned behind the Y661 and Y111 Ezh2 gating residues^35^, in a similar manner as to the arm regions of CPI-1205 and inhibitor 1^21,29^ (see discussion below). Our results, however, suggest that the tail of UNC1999, which is similar to that of GSK126, is probably also solvent exposed. Moreover, a biotinylated-tail derivative of UNC1999 is able to pull down PRC2 from cell lysates^24^, providing further support that this region is solvent exposed. While no structural information regarding the interaction of EPZ-6438 with PRC2 is available, its tail would be expected to also be solvent exposed, given a roughly similar structure to that of GSK343. This prediction is supported by the lower potency of EPZ-6438 towards the I109 and Y111 mutations of Ezh2^27,28^.

GSK126 also features a shorter *sec*-butyl arm in contrast to CPI-1205d and inhibitor 1 that both contain a long arm moiety (Fig. 1A,B and C, respectively). In PRC2 co-crystal structures of the latter two drugs, the arm regions of the drugs are trapped behind the gating residues Y661 and Y111 and point toward Eed (Fig. 5A, aligned with GSK126 structure)^21,29^. In the GSK126-bound crystal structure, the *sec*-butyl group is too short to approach Eed (Fig. 5A).

Notably, residue C663 of Ezh2 is surrounded by residue F665 as well as the indole ring of the body of GSK126, possibly mediating sulfur-π interactions (Fig. 5A and Fig. S3A). Sulfur-π interactions are prevalent in protein structures to confer stability^36^. A similar configuration was also noted for the CPI-1205d-bound structure^21^. In addition, the position of residue F665 in the structure of human PRC2-GSK126 is incompatible with SAM binding (Fig. S9A). Residue C663 of Ezh2 is replaced by S664 in Ezh1, a paralog of Ezh2, and this may account for the reported higher potency of GSK126 towards Ezh2-containing PRC2, compared to the Ezh1-containing counterpart (Fig. S9B). While a decrease in drug potency for the C663S point mutation of Ezh2 was noted for CPI-1205^21^, such a study has not been conducted for other pyridone inhibitors. We conducted IC_50_ assays with minimal PRC2-Ezh2, PRC2-Ezh1 and PRC2-Ezh2-C663S complexes. As expected, the mutant complex exhibited a 5-fold reduction of inhibition by GSK126 based on the IC_50_ values (Fig. 5B, compare to Fig. 3B). The interplay between residue C663 of Ezh2 and GSK126 awaits further examination and may lead to the development of inhibitors of a better selectivity.

## Discussion

Pyridone inhibitors are promising therapeutics for lymphoma and gaining a clearer molecular understanding of their mechanism of PRC2 inhibition will allow for the design of better future drugs. While the drugs all feature a common pyridone pharmacophore, other areas are variable and affect drug potency and specificity. In this report, we present crystal structures of GSK126 bound to human and *ct*PRC2 that are in good agreement in general. GSK126 is unique in its binding mode to Ezh2 in several regards compared to other inhibitors that have been characterized by PRC2-bound crystal structures. First, GSK126 features a long tail region that is solvent exposed and buttressed by residues of the SAL domain side of the SAL/SET gate. The “SAL gate” appears to be a dominant region for GSK126 interaction and it is at least partially functionally conserved in *ct*PRC2 (Fig. 2C) and likely other fungi (Fig. S1A). Second, the short *sec*-butyl arm of GSK126 only fills part of the pocket lying behind the SAL/SET gate, and does not approach the Ezh2-Eed interface (Fig. 5A). Our structural work suggests that GSK126 shifts the conformation of C663 (Fig. 5A) and functional work indicates that this residue plays a dominant role in inhibitor selectivity (Fig. 5B,C). Overall, the structures of PRC2 bound to GSK126 reported here reveal novel modes of drug binding that could not be conclusively deduced from docking models alone^26^.

While human and *ct*PRC2 bind to GSK126 in a similar manner, the drug has miniscule potency in *ct*PRC2. This is likely due to the absence of the SAL/SET gating residues and an ordered helix around the drug-binding site in the SET domain. These factors cause *ct*PRC2 to have a more open drug-binding site than human (Figs S4A and S4B), and likely result in poor inhibition. The drug-binding site notably represents a major structural difference between human and yeast PRC2; the SET and SAL domains differ between these organisms and we demonstrated that these differences result in disparate drug potencies. This raises the possibility that structure-based drug design could potentially be used to create optimized inhibitors that are selective for fungal PRC2 over human. For instance, engineering of the “arm” region of the drug may result in a molecule that binds better to the more open binding pocket near the SET domain of *ct*Ezh2, while being sterically hindered from binding to the more closed pocket of human Ezh2.

Finally, structural information regarding drug binding by *ct*PRC2 could be directly applicable to PRC2 from pathogenic fungi. Based on sequence alignments, PRC2 complexes from fungi are fairly conserved within the drug-binding pocket (Figs S1A and S1B). This raises the possibility that broad-spectrum PRC2 inhibitors could be developed that target fungi that are pathogenic to both humans and plants. Such inhibitors would disrupt normal gene expression programs in these pathogens. Additional research will need to be conducted to determine whether this strategy can also serve as an effective means of alleviating fungal infections.

## Materials and Methods

### Protein Expression and Purification

The wild-type PRC2 from *Chaetomium thermophilum* var. *thermophilum DSM 1495* contains a fusion of Ezh2 (residues 191–950) and the VEFS domain of Suz12 (residues 530–691), and full length Eed (residues 1–565). Ezh2-Suz12(VEFS) was co-expressed with Eed in *S*. *cerevisiae* and purified essentially as detailed previously^4,37^. Briefly, *ct*PRC2 was purified to homogeneity with IgG resin via a 2xProtein A tag on Ezh2, followed by Tobacco Etch Virus (TEV) protease cleavage of the tag, purification on StrepTactin resin (IBA Life Sciences) via a 2x StrepII tag on Eed followed by elution with d-desthiobiotin, concentration, and gel filtration on a HiLoad Superdex 200 column (GE Healthcare) in 20 mM Tris-HCl pH 8.0, 100 mM NaCl, and 2.5 mM DTT. Mutations to Ezh2 were introduced with site directed mutagenesis, and mutant proteins were expressed and purified in the same manner as wild-type *ct*PRC2.

Minimal human PRC2 complexes contained a fusion of Ezh2 (or Ezh1) to the VEFS domain of Suz12 (residues 545–695) and full length Eed (residues 1–441) and were co-expressed in *S*. *cerevisiae* and purified in a manner similar to *ct*PRC2 except without a StrepTaction column step. PRC2 used in enzymatic assays contained full length Ezh2 or Ezh1. The crystallized version of Ezh2 contained deletions of residues 183–195 and 341–42.

### Crystallization

*ct*PRC2 crystals in the apo form were produced using the published conditions^37^. Wild-type *ct*PRC2 crystals were then soaked overnight with 10 mM GSK126 in a final concentration of 20% DMSO. Humanized *ct*PRC2 crystals were soaked with GSK126 in a similar manner. After soaking, crystals were directly flash frozen in liquid nitrogen. The *hm*PRC2-GSK343 crystals were obtained by co-crystallization of protein (10 mg/mL) with 1 mM H3K27me3 peptide and 0.5 mM GSK343. Crystals were cryo-protected in well solution supplemented with 20% ethylene glycol and flash frozen in liquid nitrogen.

Human PRC2 at a concentration of 12 mg/mL was crystallized via hanging drop vapor diffusion in 200 mM ammonium citrate pH 7.2 and 14–16% PEG 3350. Crystals were soaked for 2 hours with a 5-fold molar excess of GSK126, transferred to mother solution containing 20% ethylene glycol as a cryo-protectant, and flash frozen in liquid nitrogen.

### Data Collection

Initial crystal screening was conducted at beamline 9-2 of the Stanford Synchrotron Radiation Lightsource (SSRL). Final diffraction data was collected at the Advanced Photon Source of Argonne National Laboratory at beamline 19ID on frozen crystals at −180 °C using a Pilatus detector. An oscillation angle of 0.2–0.3 was used and in total 180–360° was collected. Data was indexed, integrated, and scaled with HKL3000^38^.

### Structure Determination and Refinement

All structures were solved by molecular replacement with PHASER in the PHENIX software suite^39,40^. *ct*PRC2 and *hm*PRC2 structures used the apo complex (PDB 5bjs) as a search model. The human PRC2 structure used the *h/Ac*PRC2 structure bound to inhibitor 1 (PDB 5ij7) as a search model with the drug deleted for the search. Structures then underwent manual building and adjustment using the program Coot^41^. Ligand restraints were generated with the program Elbow of the PHENIX suite^42^. Drugs were fit into Fo − Fc electron density using the ligand finder option in Coot. Structures were refined with Refmac, autoBuster, and PHENIX using torsion, libration, and screw (TLS) refinement^39,43,44^.

The *ct*PRC2 and *hm*PRC2 structures each contained one complex per asymmetric unit. Human PRC2 contained two complexes per asymmetric unit. One complex, however, had several regions that were more poorly ordered compared to the other. Therefore, the structure underwent extensive, manual truncation to remove regions from the search model that were missing from the electron density. GSK126 could only be fit into Ezh2 from one complex, likely due to the poorer order of the second PRC2 in the asymmetric unit. The construct for human PRC2 contained a mutation of Ezh2 residue W594R that was inadvertently introduced during cloning. Crystals could be obtained of human PRC2-GSK126 with the wild-type sequence, but diffraction quality was not improved (data not shown).

### IC_50_ Analysis

For assays with human PRC2, 50 nM of PRC2 was mixed with 1 μM of histone H3K27me0 peptide (residues 22–44, AnaSpec catalogue number AS-64641), and a titration of GSK126 (dissolved in 100% DMSO) in buffer containing 25 mM Tris-HCl pH 8.0, 10 mM NaCl, 1 mM EDTA, 2.5 mM MgCl_2_, and 5 mM DTT in a 20 μL reaction volume. Reactions contained a final concentration of 2% DMSO. A final concentration of 5 μM SAM was used per reaction at a ratio of 1: 17 unlabeled to ^3^H SAM labeled SAM (specific activity of 82.5–85 Ci/mmol, final concentration of 0.33 μM; Perkin Elmer). Reactions containing *ct*PRC2 and *hm*PRC2 were prepared in a similar manner except they contained 15 nM enzyme and no unlabeled SAM. After 1 hour incubation at 30 °C, reactions were stopped by the addition of 1.1 mM unlabeled SAM. An amount of 10 μL of the reaction was then spotted onto phosphocellulose filters (Reaction Biology Corporation) and dried. Filters were washed 5 times with 50 mL of 50 mM NaCO_3_/NaHCO_2_ pH 9.0, rinsed with acetone, dried, and immersed in 4 mL of scintillation fluid. Filters were counted with a scintillation counter with read-out in disintegrations per minute (DPM). Experimental values were subtracted from a control sample that contained enzyme but no peptide substrate. Assays used at least eight titration points and all points were performed in duplicate. Curve-fitting and IC_50_ value determination was calculated with GraphPad Prism 7 using log(inhibitor) vs. response analysis. IC_50_ of GSK343 for wild type *ct*PRC2 and hmPRC2 was measured essentially the same as described above, except that 59 nM enzyme complex was used.

### Steady-state enzyme kinetics

Kinetic experiments were performed with a titration of H3K27me0 peptide (Anaspec, AS-64440-1) in a manner similar to that described previously^33^. Briefly, assay buffer contains 25 mM Tris pH 8.0, 10 mM NaCl, 1 mM EDTA, 2.5 mM MgCl_2_ and 5 mM DTT. 59 nM PRC2 was used for each reaction in the presence of 20 μM SAM with different concentration of H3K27me0 peptide titrated in. The reaction was left to proceed for 1 hour at 30 °C before it was stopped by cold SAM.

### Accession Numbers

Coordinates and structure factors for the structures reported here were deposited in the Protein Data Bank under the following accession codes: 5wf7 (*ct*PRC2 bound to GSK126), 5wg6 (human PRC2 bound to GSK126), 5wfd (*hm*PRC2 bound to GSK126), and 5wfc (*hm*PRC2 bound to GSK343).

## Electronic supplementary material

Supplementary Information
