# Supplementary material for: An Evolutionarily Conserved Structural Platform for PRC2 Inhibition by a Class of Ezh2 Inhibitors
Source: Sci Rep. 2018 Jun 14;8:9092. doi: 10.1038/s41598-018-27175-w (PMC6002473; doi:10.1038/s41598-018-27175-w)
Supplement: Supplementary file 1 — Supplementary Information [file 41598_2018_27175_MOESM1_ESM.pdf]

Supplementary Information

An Evolutionarily Conserved Structural Platform for PRC2 Inhibition by a Class of Ezh2 Inhibitors

Matthew Bratkowski, Xin Yang and Xin Liu *

Cecil H. and Ida Green Center for Reproductive Biology Sciences and Division of Basic Research, Department of Obstetrics and Gynecology
Department of Biophysics
UT Southwestern Medical Center, Dallas, TX 75390, USA.

[*] Corresponding author. E-mail: xin.liu@utsouthwestern.edu. Phone: (214) 648-2493. Fax: (214) 648-0383.

Supplemental Figure Legends

Figure S1. Sequence alignment of Ezh2 proteins from several fungal species and human. The SAL domain (*magenta*) and SET domain (*blue*) are outlined. Green boxes above the alignment represent residues important for GSK126 binding in *ct*PRC2 and red boxes below the alignment represent residues important for GSK126 binding in human PRC2. Abbreviations are as follows: *ct*: *Chaetomium thermophilum; nc: Neurospora crassa; fg: Fusarium graminearum; cn: Cryptococcus neoformans; h: human.*

Figure S2. SDS-PAGE purity analysis of PRC2 complexes used in this study. A) Lanes 1 and 2, wildtype and humanized (*hm*) *ct*PRC2 complexes, *respectively*. Lane 3, molecular weight marker (in KDa). Lane 4 and 5, full-length human Ezh2-PRC2 and crystallized truncated Ezh2-PRC2, *respectively*. B) Human PRC2 complexes used for IC_{50} assays.

Figure S3. Electron density quality of the drug-binding pocket of the human PRC2-GSK126 bound structure. Ezh2 residues are shown as green cartoon with side-chains as sticks, and GSK126 is in yellow sticks. 2Fo-Fc electron density is contoured at 1σ and shown as black mesh for Ezh2 and cyan mesh for GSK126.

Figure S4. Surface representations of PRC2 drug binding pockets. Ezh2 is in gray, Eed in green, and Suz12 in cyan. Drugs are in sticks of various colors. A)

Human PRC2-GSK126 bound structure. B) *ct*PRC2 wildtype-GSK126 structure. C) *hm*PRC2-GSK126 structure. D) *hm*PRC2-GSK343 structure.

Figure S5. Detailed views of the SET (*blue sticks*) and SAL (*magenta sticks*) regions of *ct*Ezh2 and *hm*Ezh2 around the drug-binding pocket. Drugs are shown as sticks. 2Fo-Fc, contoured at 1σ, encloses protein residues (*black mesh*) and drugs (*cyan mesh*). A) *ct*PRC2 wildtype-GSK126 structure. B) *hm*PRC2-GSK126 structure. C) *hm*PRC2-GSK343 structure.

Figure S6. Steady-state enzyme kinetic analysis of wild-type and humanized mutant *ct*PRC2. A saturating concentration of 20μM SAM and a titration of histone H3K27me0 peptide were used for the assays.

Figure S7. Dose-response curves of wildtype (*left*) and humanized (*right*) *ct*PRC2 under a titration of GSK343. Note that the solubility of GSK343 was limited. Wild-type *ct*PRC2 never reached significant inhibition even at high concentrations of drug and therefore the IC_{50} value calculated represents a rough estimate.

Figure S8. Detailed view of the *hm*PRC2 and *ct*PRC2 stimulating peptide binding pocket with Eed (*green cartoon*), the SRM region (*pink cartoon*), H3K27me3 peptide (*yellow sticks*), and Suz12(VEFS) (*cyan*).

A) *hm*PRC2-GSK343 structure. The stimulating H3K27me3 peptide is enveloped by 2Fo-Fc electron density (*blue mesh*) contoured at 1σ.

B) *ct*PRC2-stimulated state structure (PDB 5kkl).

Figure S9. A) Structural aligment-comparison of residues in the SET domain of the human PRC2-GSK126 structure (*gray*) with SAH (*orange sticks*) and K27M peptide (*magenta*) from the stimulated state human PRC2 structure (PDB 5hyn).

B) Sequence alignment of human Ezh2 and Ezh1 SET domain residues around the drug-binding site. C663 in Ezh2 is highlighted with a green triangle above the sequence.

Fig. S1

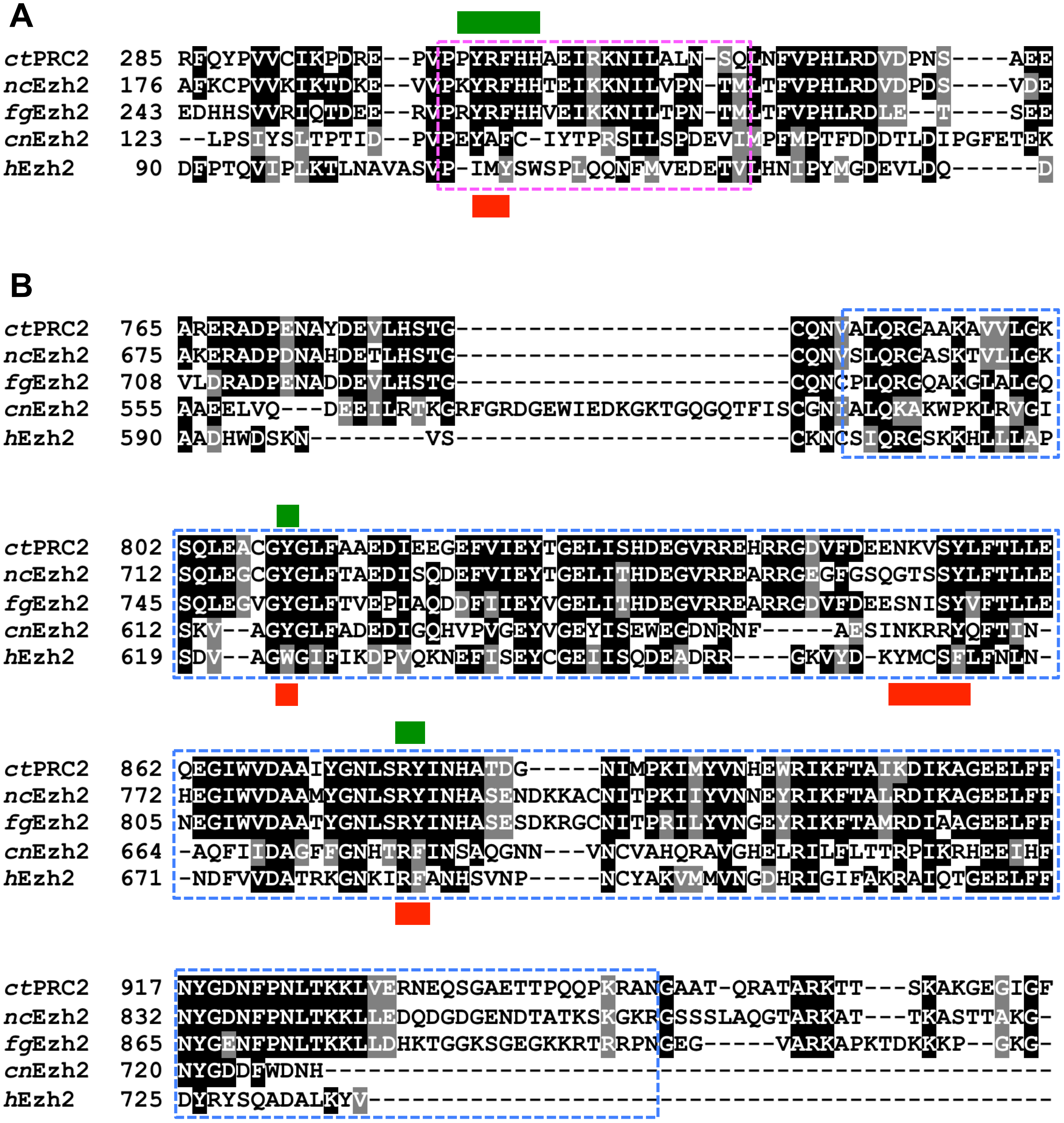
A
ctPRC2 285 RFQYPVVCIKPDRE--PVPPYRFHHAEIRKNILALN-SQLNFVPHLRDVDPNS----AEE
ncEzh2 176 AFKCPVVKIKTDKE--VVPKYRFHHTEIKKNILVPN-TMLTFVPHLRDVDPDS----VDE
fgEzh2 243 EDHHSVVRIQTDEE--RVPRYRFHHVEIKKNILTPN-TMLTFVPHLRDLE--T----SEE
cnEzh2 123 --LPSIYSLTPTID--PVPEYAFC-IYTPRSILSPDEVIMPFMPTFDDDTLDIPGFETEK
hEzh2 90 DFPTQVIPLKTLNAVASVP-IMYSWSPLQQNFMVEDETVLHNIPYMGDEVLDQ------D
B
ctPRC2 765 ARERADPENAYDEVLHSTG-----------------------CQNVALQRGAAKAVVLGK
ncEzh2 675 AKERADPDNAHDETLHSTG-----------------------CQNVSLQRGASKTVLLGK
fgEzh2 708 VLDRADPENADDEVLHSTG-----------------------CQNCPLQRGQAKGLALGQ
cnEzh2 555 AAEELVQ---DEEILRTKGRFGRDGEWIEDKGKTGQGQTFISCGNIALQKAKWPKLRVGI
hEzh2 590 AADHWDSKN--------VS-----------------------CKNCSIQRGSKKHLLLAP
ctPRC2 802 SQLEACGYGLFAAEDIEEGEFVIEYTGELISHDEGVRREHRRGDVFDEENKVSYLFTLLE
ncEzh2 712 SQLEGCGYGLFTAEDISQDEFVIEYTGELITHDEGVRREARRGEGFGSQGTSSYLFTLLE
fgEzh2 745 SQLEGVGYGLFTVEPIAQDDFIIEYVGELITHDEGVRREARRGDVFDEESNISYVFTLLE
cnEzh2 612 SKV--AGYGLFADEDIGQHVPVGEYVGEYISEWEGDNRNF-----AESINKRRYQFTIN-
hEzh2 619 SDV--AGWGIFIKDPVQKNEFISEYCGEIISQDEADRR----GKVYD-KYMCSFLFNLN-
ctPRC2 862 QEGIWVDAAIYGNLSRYINHATDG-----NIMPKIMYVNHEWRIKFTAIKDIKAGEELFF
ncEzh2 772 HEGIWVDAAMYGNLSRYINHASENDKKACNITPKIIYVNNEYRIKFTALRDIKAGEELFF
fgEzh2 805 NEGIWVDAATYGNLSRYINHASESDKRGCNITPRILYVNGEYRIKFTAMRDIAAGEELFF
cnEzh2 664 -AQFIIDAGFFGNHTRFINSAQGNN---VNCVAHQRAVGHELRILFLTTRPIKRHEEIHF
hEzh2 671 -NDFVVDATRKGNKIRFANHSVNP-----NCYAKVMMVNGDHRIGIFAKRAIQTGEELFF
ctPRC2 917 NYGDNFPNLTKKLVERNEQSGAETTPQQPKRANGAAT-QRATARKTT---SKAKGEGIGF
ncEzh2 832 NYGDNFPNLTKKLLEDQDGDGENDTATKSKGKRGSSSLAQGTARKAT---TKASTTAKG-
fgEzh2 865 NYGENFPNLTKKLLDHKTGGKSGEGKKRTRRPNGEG-----VARKAPKTDKKKP--GKG-
cnEzh2 720 NYGDDFWDNH--
hEzh2 725 DYRYSQADALKYV---

Fig. S2

A

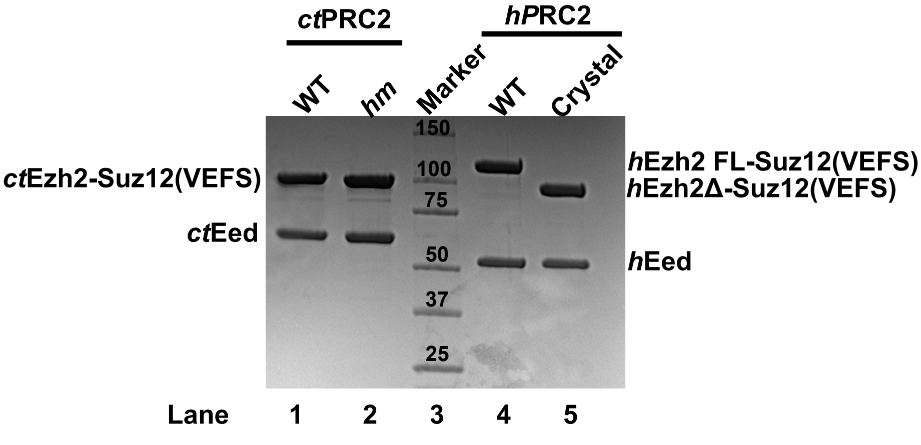
ctPRC2
hPRC2
WT
hm
Marker
WT
Crystal
150
100
75
50
37
25
ctEzh2-Suz12(VEFS)
ctEed
hEzh2 FL-Suz12(VEFS)
hEzh2Δ-Suz12(VEFS)
hEed
Lane 1 2 3 4 5

B

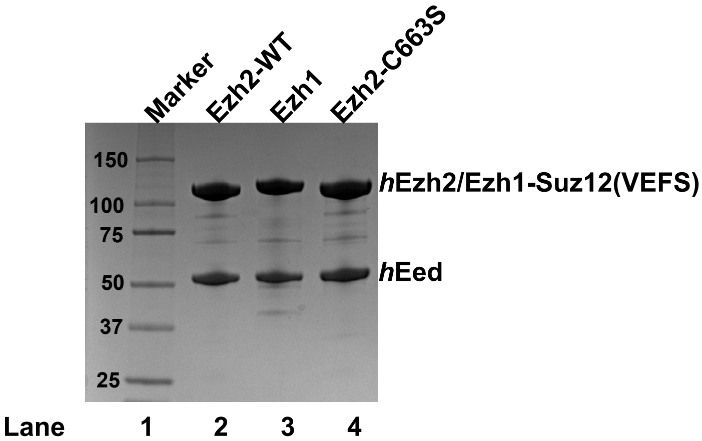
Marker
Ezh2-WT
Ezh1
Ezh2-C663S
150
100
75
50
37
25
hEzh2/Ezh1-Suz12(VEFS)
hEed
Lane 1 2 3 4

Fig. S3

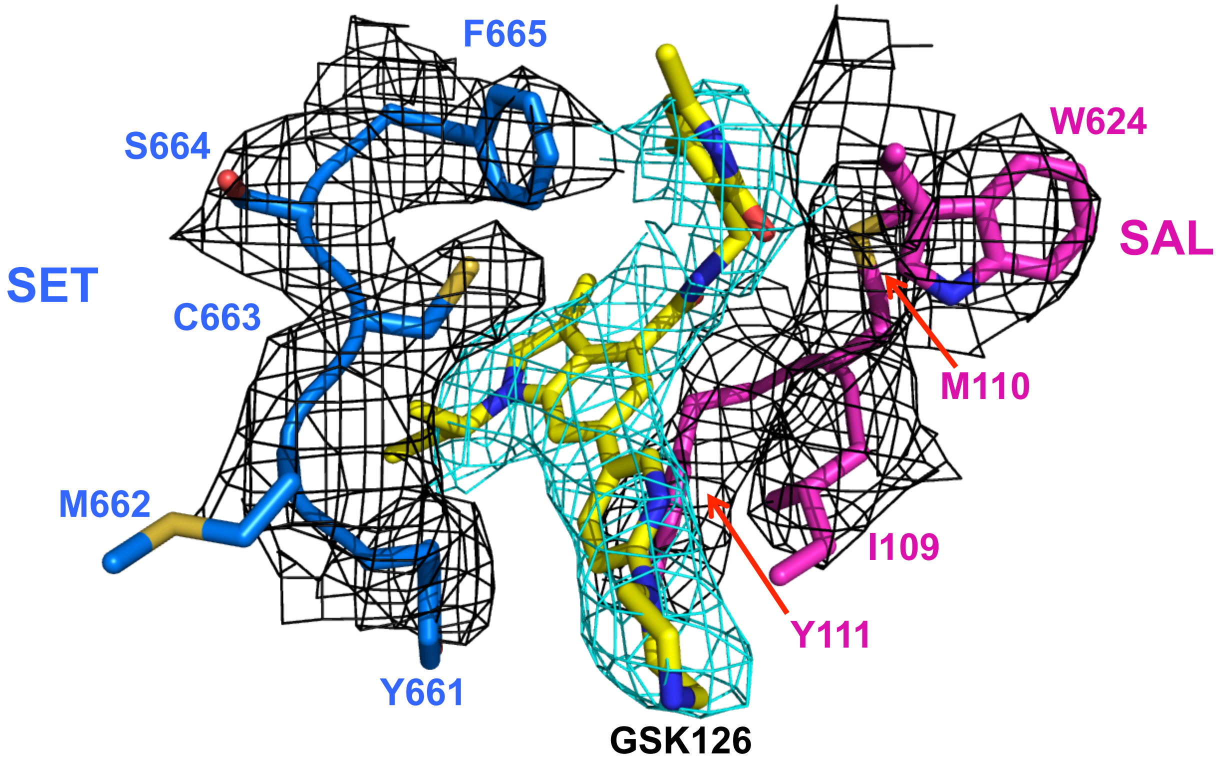

Fig. S4

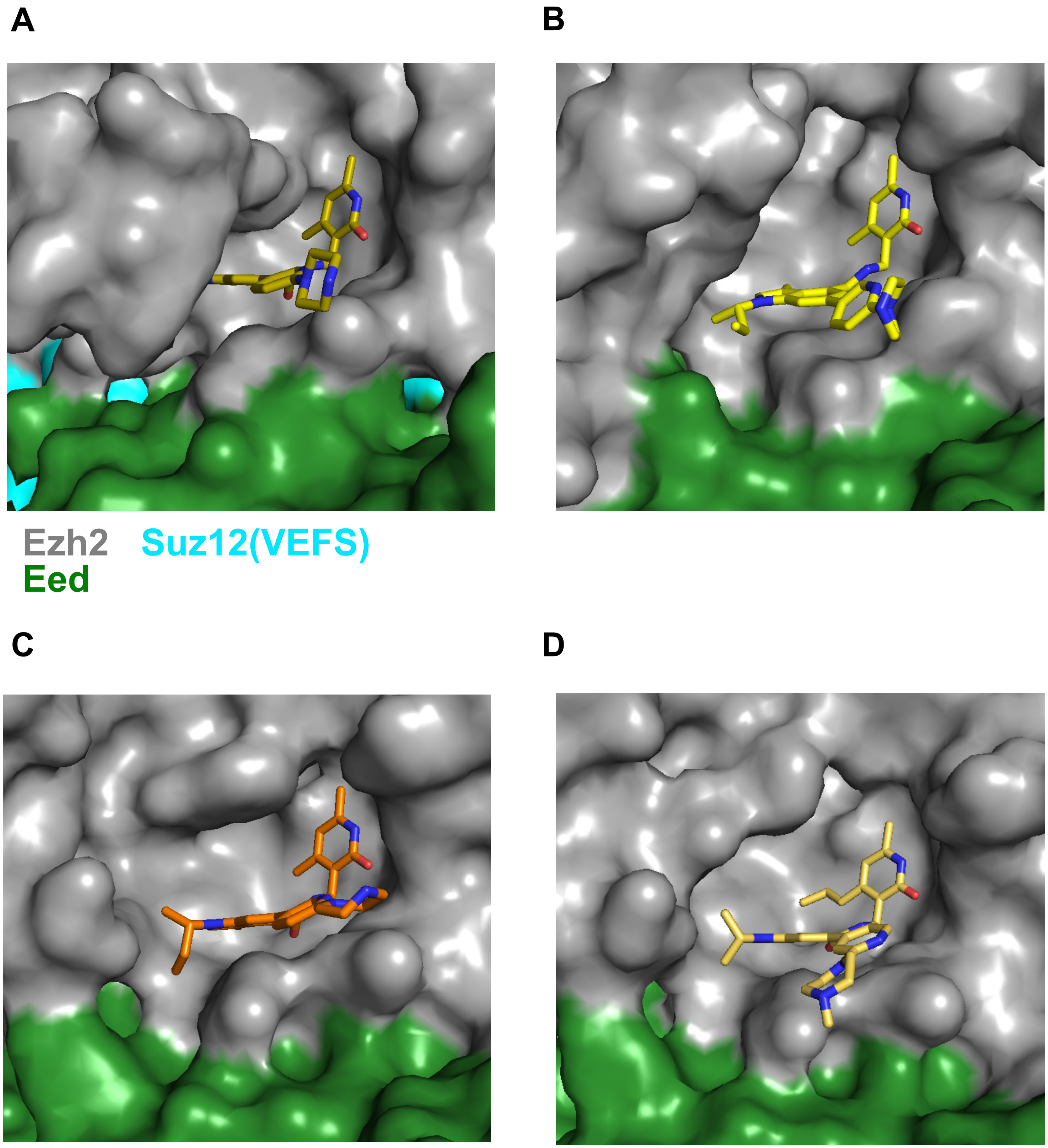

Fig. S5

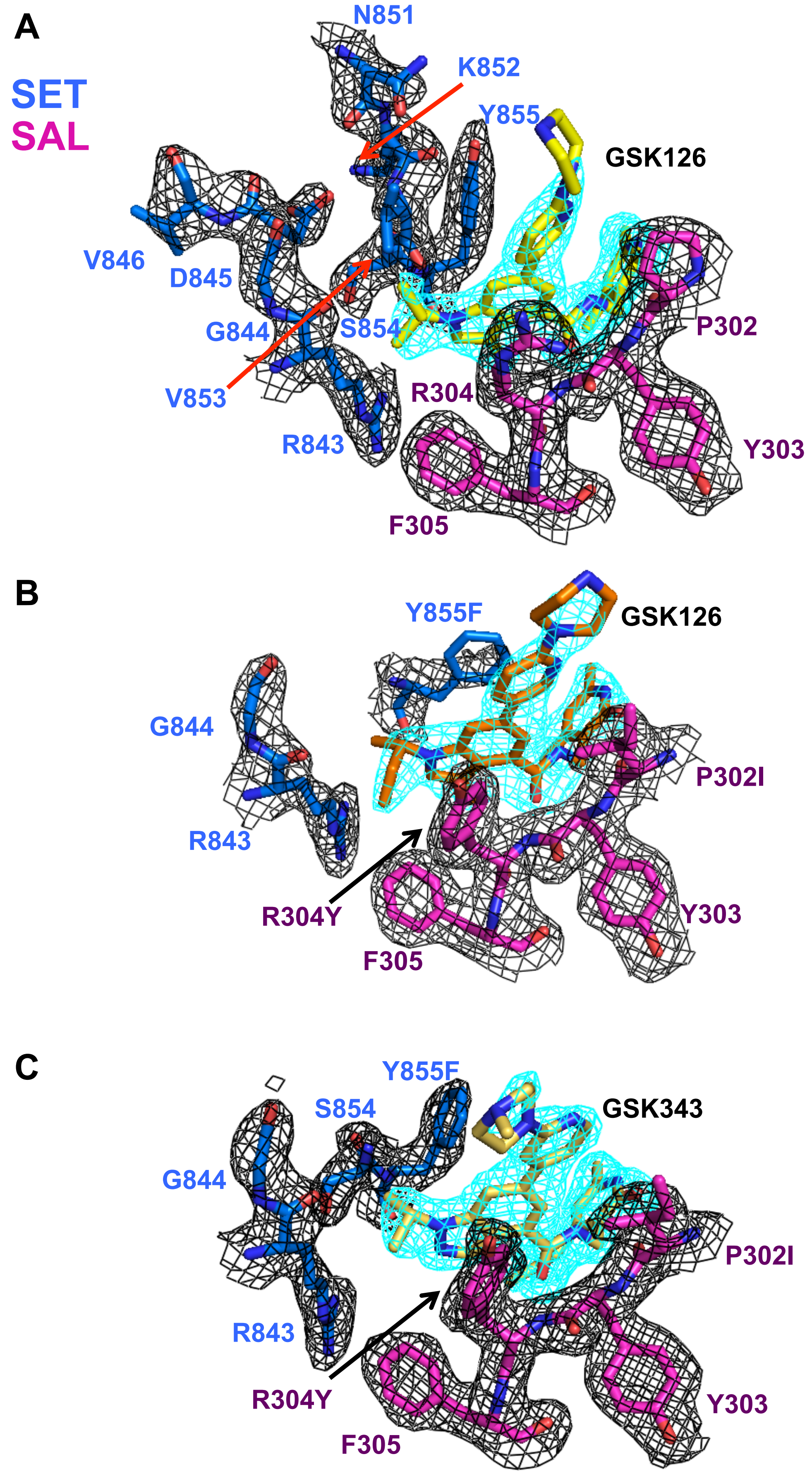
A
SET
SAL
N851
K852
Y855
GSK126
V846
D845
G844
S854
P302
V853
R304
R843
Y303
F305
B
Y855F
GSK126
G844
P302I
R843
R304Y
Y303
F305
C
Y855F
S854
GSK343
G844
P302I
R843
R304Y
Y303
F305

Fig. S6

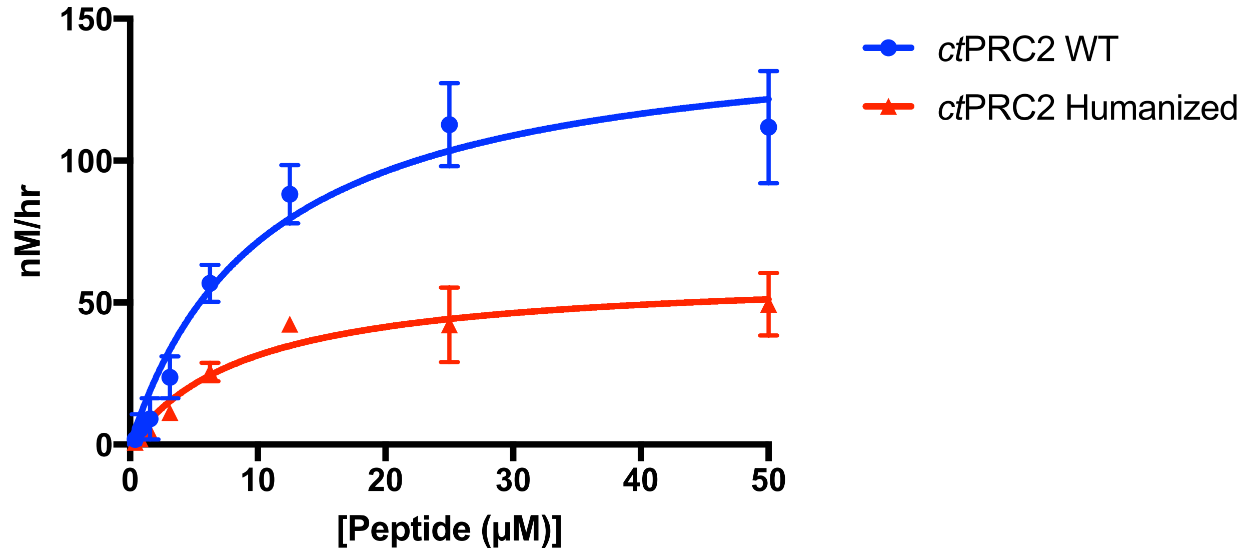

*ct*PRC2 Version	Km (μM)	kcat (hr^{-1})
WT	10.7 ± 2.2	2.6 ± 0.2
Humanized	9.3 ± 2.5	1.1 ± 0.1

Fig. S7

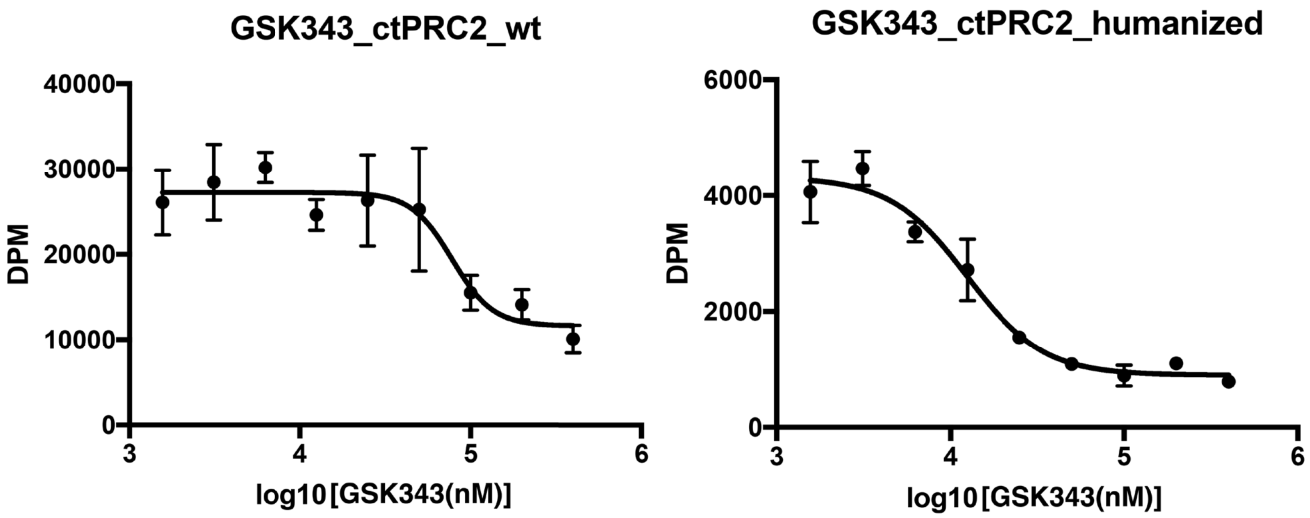

*ct*PRC2 Version	IC_{50} (μM)	95% Confidence Intervals (μM)
WT	78.0	55.1 to 99.5
Humanized	12.5	9.6 to 15.8

Fig. S8

A

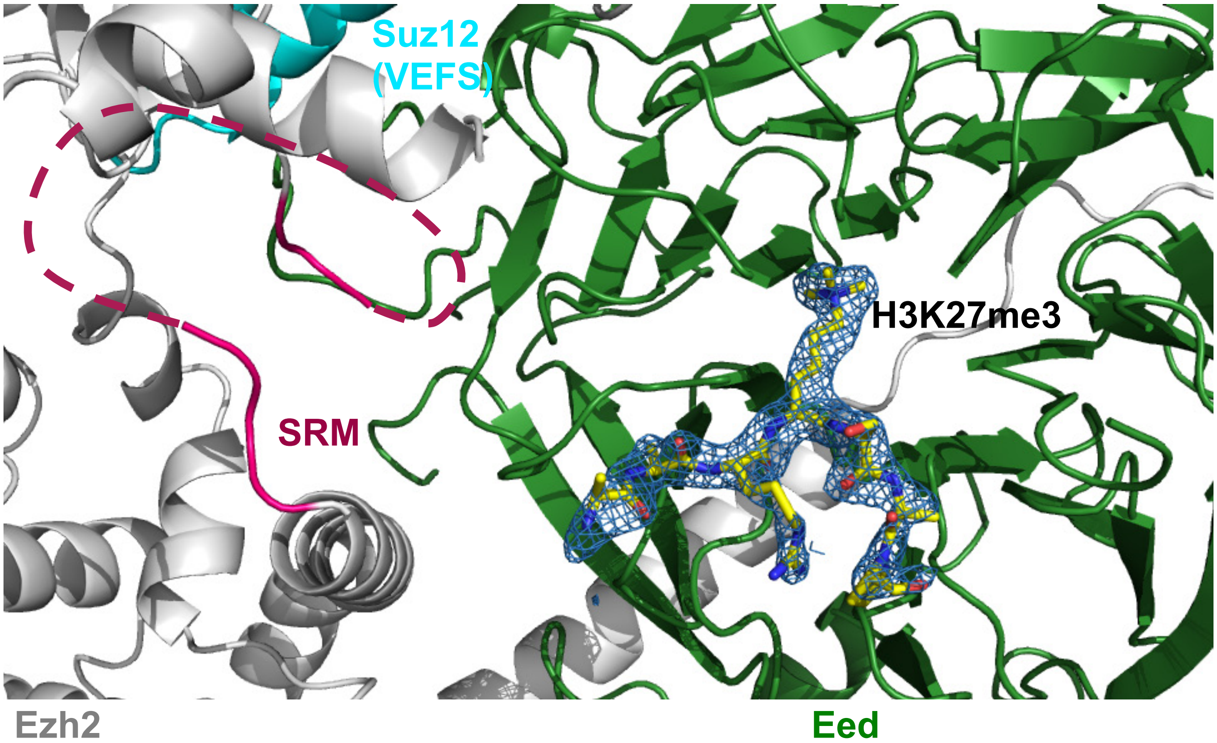
Suz12
(VEFS)
H3K27me3
SRM
Ezh2
Eed

B

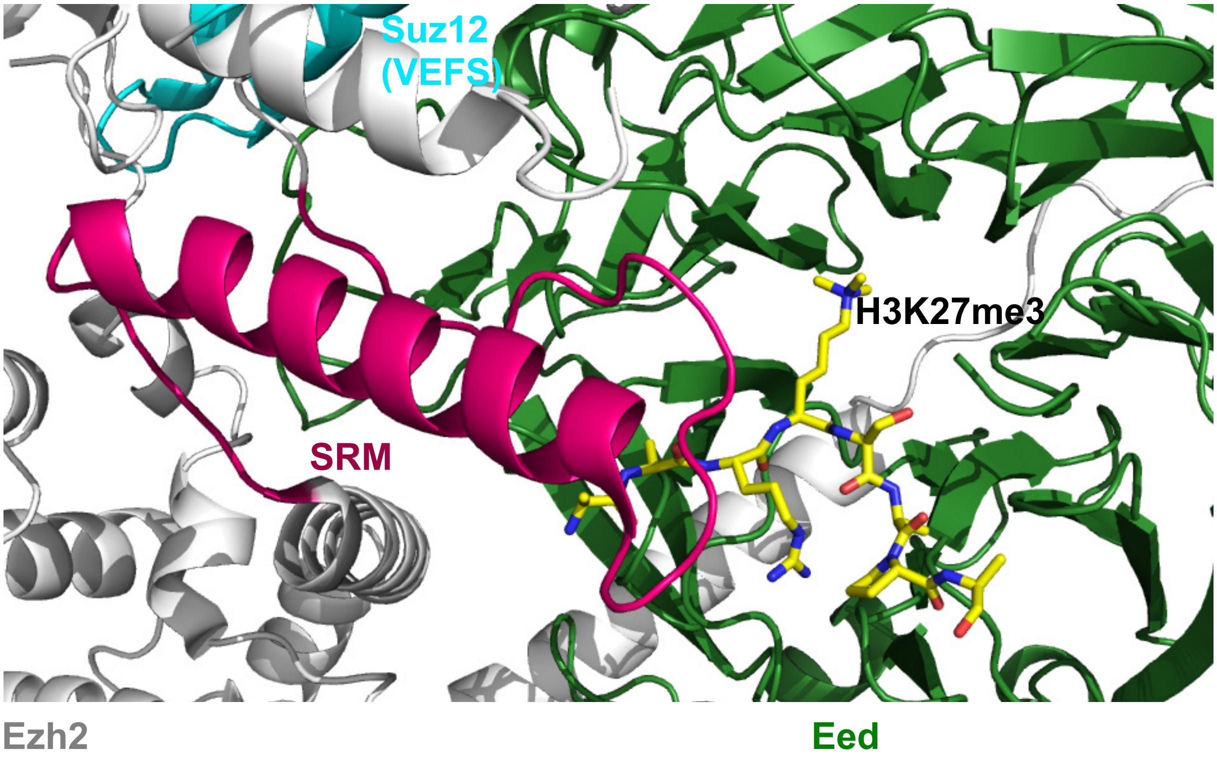
Suz12
(VEFS)
H3K27me3
SRM
Ezh2
Eed

Fig. S9

A

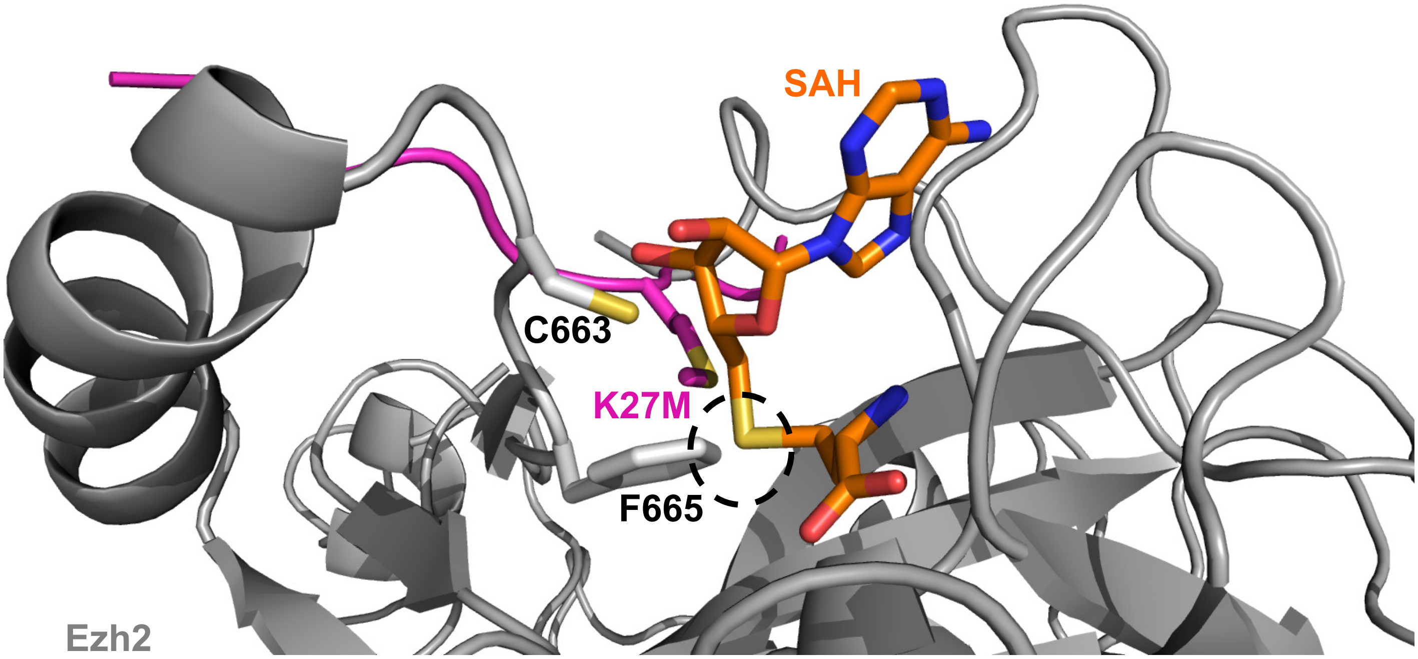

B

```
EZH2   600  SCKNCSIQRGSKKHLLLAPSDVAGWGIFIKDPVQKNEFISEYCGEIISQDEADRRGKVYD
EHZ1   601  SCKNCSIQRGLKKHLLLAPSDVAGWGTFIKESVQKNEFISEYCGELISQDEADRRGKVYD

EZH2   660  KYMCSFLFNLNNDFVVDATRKGNKIRFANHSVNPNCYAKVMMVNGDHRIGIFAKRAIQTG
EHZ1   661  KYMSSFLFNLNNDFVVDATRKGNKIRFANHSVNPNCYAKVVMVNGDHRIGIFAKRAIQAG

EZH2   720  EELFFDYRYSQADALKYVGIEREMEIP
EHZ1   721  EELFFDYRYSQADALKYVGIERETDVL
```

Table 1 Data collection and refinement statistics (molecular replacement)

Crystal<br>PDB ID	*ct*PRC2-GSK126<br>5WF7	*h*PRC2-GSK126<br>5WG6	*hm*PRC2-GSK126<br>5WFD	*hm*PRC2-GSK343<br>5WFC
**Data collection**[a]				
Space group	$C222_1$	I422	$C222_1$	$C222_1$
Cell dimensions				
a, b, c (Å)	115.9, 136.3, 223.2	243.9, 243.9, 243.7	117.5, 137.0, 223.6	117.6, 137.8, 222.3
α, β, γ (°)	90, 90, 90	90, 90, 90	90, 90, 90	90, 90, 90
Resolution (Å)	50 – 2.50 (2.56 – 2.50)[b]	50 – 3.90 (3.97 – 3.90)	50 – 2.65 (2.70 – 2.65)	50 – 2.29 (2.33 – 2.29)
R_{merge}	0.119 (1.896)	0.375 (4.734)	0.213 (3.146)	0.109 (1.126)
R_{pim}	0.035 (0.562)	0.075 (0.939)	0.066 (0.975)	0.033 (0.454)
$I / \sigma I$	22.5 (1.56)	14.6 (1.17)	14.6 (1.78)	24.6 (2.11)
$CC_{1/2}$	0.896 (0.541)	0.932 (0.646)	0.882 (0.535)	0.925 (0.476)
Completeness (%)	100 (100)	100 (100)	100 (100)	99.1 (91.4)
Redundancy	12.6 (12.1)	26.2 (26.2)	11.4 (11.1)	12.0 (7.1)
**Refinement**				
Resolution (Å)	44.1 – 2.50 (2.56 – 2.50)	40.6 – 3.90 (3.97 – 3.90)	46.1 – 2.65 (2.70 – 2.65)	44.7 – 2.29 (2.33 – 2.29)
No. reflections	57672	26797	50218	79297
R_{work} / R_{free}	0.187/0.258	0.235/0.300	0.178/0.239	0.161/0.203
No. atoms	10325	13474	10128	10898
Protein	9972	13419	10081	10164
Inhibitor	39	39	39	40
Zinc	8	16	8	8
Water	306	0	0	686
Ave. B-factors (Å^2)				
Protein	52.18	50.79	50.45	42.16
Ligand/ion	52.05	40.58	43.26	46.18
Water	41.35	0	0	42.25
R.m.s. deviations				
Bond lengths (Å)	0.011	0.017	0.012	0.009
Bond angles (°)	1.067	0.663	1.075	0.888
Ramachandran Plot				
Favored (%)	95.50	87.28	94.90	96.20
Allowed (%)	4.01	11.53	4.94	3.72
Outlier (%)	0.49	1.18	0.16	0.08

[a]Data were collected from a single crystal. [b]Values in parentheses are for highest-resolution shell.
